# Space Radiation and Cancer Risk in Astronauts: Models, Evidence, Uncertainties, and Emerging Imaging Perspectives

**DOI:** 10.3390/tomography12070106

**Published:** 2026-07-17

**Authors:** Chiara Zanon, Michele Basilicata, Agostino Chiaravalloti, Nicola Giannotti, Amalia Lupi, Filippo Crimì, Emilio Quaia

**Affiliations:** 1 Department of Radiology, University of Padova, Via Giustiniani 2, 35128 Padova, Italy; 2 Radiology Department, UniCamillus-Saint Camillus International University of Health Sciences, 00131 Rome, Italy; 3 UOSD Special Care Dentistry, Department of Experimental Medicine and Surgery, University of Roma Tor Vergata, 00133 Rome, Italy; 4 Department of Biomedicine and Prevention, University of Rome Tor Vergata, Viale Montpellier 1, 00133 Rome, Italy; 5 IRCCS Neuromed Mediterranean Neurological Institute, Via Atinense 18, 86077 Pozzilli, Italy; 6 Faculty of Medicine and Health, The University of Sydney, Sydney, NSW 2006, Australia

**Keywords:** space radiation, astronauts, cancer risk estimation, galactic cosmic rays, REID, high-LET radiation, quantitative imaging, imaging biomarkers

## Abstract

Cancer risk estimation remains one of the main unresolved challenges in human spaceflight beyond low Earth orbit, where astronauts are exposed to galactic cosmic rays, solar particle events, and high-linear energy transfer (high-LET) secondary radiation. This narrative review summarizes the principal quantitative models used to estimate radiation-induced cancer risk in astronauts, including particle fluence-based cross-sections, mixture models, risk of exposure-induced death (REID)-based operational frameworks, uncertainty distribution approaches, and ensemble models. Early studies estimated 1-year excess cancer mortality at solar minimum as 1.3% in women and 1.1% in men under 10 g/cm^2^ aluminum shielding, whereas later models projected non-leukemia lifetime cancer incidence after 1 Sv dose equivalent/effective dose between 2.20% and 2.98%, depending on sex and age. Earlier REID-based models suggested that the historical 3% REID threshold could be exceeded after approximately 18 months in women and 24 months in men under unfavorable solar conditions, whereas the current NASA radiation standard uses a universal career-effective dose limit of 600 mSv, applied regardless of sex or age. More recent revisions of the NASA Space Cancer Risk model and non-targeted effect scenarios suggest that exploration mission risks may be higher than previously estimated, while uncertainty remains substantial, especially for high-LET radiobiology, mixed-field exposure, and the transfer of terrestrial epidemiological data to the spaceflight setting. Future progress may also involve exploring quantitative imaging biomarkers and tomographic assessments as complementary tools for longitudinal monitoring and early detection of radiation-related tissue changes, although these approaches are not yet validated as components of operational astronaut cancer risk models.

## Simple Summary

Astronauts who travel beyond low Earth orbit are exposed to space radiation that is very different from most radiation on Earth. This radiation may increase the long-term risk of cancer, but estimating this risk is difficult because direct human data are limited and current models rely largely on information from terrestrial radiation studies. This review summarizes the main models used to estimate cancer risk in astronauts and explains why important uncertainties remain, especially for high-energy particles, mixed radiation fields, mission duration, sex, age, and shielding. The review also discusses how tomographic imaging techniques, such as CT, MRI, PET, and radiomics, may in the future help monitor radiation-related tissue changes and support more personalized astronaut health surveillance. However, these imaging approaches are still exploratory and are not yet validated as part of operational astronaut cancer risk models.

## 1. Introduction

Human space exploration beyond low Earth orbit exposes astronauts to radiation environment that differs substantially from that encountered on Earth [1]. Outside the protection of the geomagnetic field, crews are chronically exposed to galactic cosmic rays, solar particle events, and secondary radiation generated within spacecraft shielding and biological tissues [2]. Secondary radiation comprises multiple particle types with broad energy and LET distributions, including low- and high-LET components. This mixed field includes high-linear energy transfer particles, such as heavy ions, whose biological effectiveness is greater and more uncertain than that of conventional low-LET terrestrial radiation [3]. Radiation-induced cancer remains one of the major limiting factors for long-duration missions to the Moon, Mars, and other deep-space destinations [3,4]. Estimating cancer risk in astronauts is particularly challenging because direct human data are limited, and current models largely depend on the transfer of terrestrial epidemiological evidence, especially from the Life Span Study of atomic bomb survivors, to the spaceflight setting [5]. However, this extrapolation is affected by major uncertainties related to dose rate, radiation quality, shielding, sex, age at exposure, and mission duration [6]. Earlier particle-based and transfer models provided the first quantitative estimates of astronaut cancer risk, as summarized in Section 6.1 and Table 1 [7]. Earlier NASA REID-based modeling suggested that the historical 3% REID threshold could be exceeded after approximately 18 months in women and 24 months in men under unfavorable solar conditions [8,9]. However, these values should be interpreted as model-based estimates from the previous sex- and age-specific REID framework, not as current operational mission duration limits. NASA’s current radiation exposure standard uses a universal career-effective dose limit of 600 mSv for spaceflight radiation exposure, applied regardless of sex and age. This change simplifies the older sex- and age-dependent framework while maintaining a dose limit intended to protect against radiation-induced cancer risk [10,11].

Over the past decades, cancer risk modeling for astronauts has evolved from particle fluence-based cross sections to probabilistic and ensemble approaches that incorporate uncertainty in radiation quality factors, dose and dose-rate effectiveness, excess risk transfer, and latency [12]. Emerging evidence on non-targeted effects suggests that conventional models may underestimate deep-space oncological risk, potentially increasing projected fatal cancer risk by two- to fourfold in some exploration scenarios [13].

The aim of this narrative review is to examine the main approaches used to estimate cancer risk in astronauts exposed to space radiation, summarize the principal quantitative findings, and discuss the major sources of uncertainty that still limit accurate prediction for future exploration missions. A specific objective is to clarify how tomographic imaging may realistically contribute to future astronaut monitoring and radiation-related risk assessment while distinguishing established evidence from exploratory applications. Beyond its relevance to space medicine, this topic is also of interest to the imaging community because Future astronaut monitoring may increasingly benefit from exploratory quantitative imaging biomarkers.

In this perspective, understanding how current risk models are constructed and where their uncertainties arise may inform the development of imaging-based approaches for early detection, longitudinal monitoring, and personalized assessment of radiation-induced oncologic risk.

However, the role of tomographic imaging in astronaut cancer risk assessment remains insufficiently defined. Current risk models rely mainly on dosimetry, radiobiology, and epidemiological risk transfer, while imaging-derived biomarkers remain outside operational prediction frameworks [14]. This gap is important for the imaging community because CT, MRI, PET, and radiomics could provide quantitative longitudinal data on radiation-related tissue changes beyond population-based models [14]. By reviewing current cancer risk models, this article highlights opportunities for tomographic imaging in future personalized surveillance, early detection, and risk refinement strategies [14].

## 2. Space Radiation Environment

The space radiation environment is a complex mixed field composed primarily of galactic cosmic rays (GCRs), solar particle events (SPEs), and secondary radiation generated by interactions with spacecraft shielding and human tissues [15]. A key feature of this environment is the coexistence of low-linear energy transfer (low-LET) and high-linear energy transfer (high-LET) radiations. LET describes the amount of energy deposited by ionizing radiation per unit track length in tissue. Low-LET radiation, such as X-rays, gamma rays, and protons, deposits energy sparsely along its track and generally produces more isolated DNA lesions. In contrast, high-LET radiation, including heavy ions and high-charge and high-energy (HZE) nuclei, deposits energy densely over very short distances, producing clustered DNA damage, complex chromosomal aberrations, and biological effects that are more difficult for cells to repair. High-LET particles generally have a higher relative biological effectiveness (RBE) than conventional low-LET radiation and are more difficult to shield effectively [16].

GCRs are dominated by protons (~85%), alpha particles (~14%), and a smaller fraction of HZE nuclei (~1%), but HZE particles contribute disproportionately to biological damage because of their high ionization density and complex track structure [17]. In radiation risk models, these physical and biological differences are incorporated through parameters such as the quality factor (QF), which weighs the absorbed dose according to radiation quality, and the dose and dose-rate effectiveness factor (DDREF), which accounts for differences between high-dose-rate exposures and chronic low-dose-rate exposures that are more typical of spaceflight. Defining these terms early is important because QF and DDREF strongly influence cancer risk projections and represent major sources of uncertainty in current models of astronaut risk.

Radiation exposure is also strongly influenced by the solar cycle phase: during the solar minimum, the GCR flux increases, leading to higher cumulative doses and greater projected cancer risk. Earlier models estimated 1-year excess cancer mortality under 10 g/cm^2^ aluminum shielding at solar minimum as 1.3% in women and 1.1% in men [18]. This dosimetric and radiobiological complexity, combined with variable shielding effectiveness and mixed particle exposure, makes accurate oncologic risk projection particularly challenging [19].

## 3. Main Approaches to Cancer Risk Estimation

In this review, imaging perspectives are considered emerging complementary tools rather than established components of current operational risk models.

The main approaches to astronaut cancer risk estimation have evolved from deterministic particle-based calculations to probabilistic and mission-specific models [20]. Early methods used risk cross-sections per particle fluence, converting organ-specific LET spectra into estimates of excess cancer mortality and lifetime cancer incidence, with substantial uncertainty related to shielding, radiation quality, sex, and age at exposure [21].

Using this framework, the lifetime non-leukemia cancer incidence after 1 Sv dose equivalent/effective ranged from 2.20% to 2.98%, depending on sex and age at exposure [21]. NASA subsequently formalized these methods in the Space Cancer Risk (NSCR) model, whose principal output is the risk of exposure-induced death (REID) with age- and sex-specific uncertainty bounds [20].

It is important to distinguish between the different risk endpoints used across studies. Excess cancer mortality refers to the additional cancer deaths attributable to radiation exposure, whereas lifetime cancer incidence refers to the probability of developing cancer over the remaining lifetime. Fatal cancer risk and REID are mortality-based endpoints, whereas the probability of causation estimates the likelihood that a cancer occurring in an exposed individual is attributable to radiation exposure.

These endpoints are related but not interchangeable with each other. Similar model-dependent variability has also been reported in terrestrial diagnostic-exposure settings. Quaia et al. showed that BEIR VII, ICRP 103, and US EPA models produced significantly different oncogenic risk estimates in ICU patients exposed to repeated diagnostic ionizing radiation, despite good-to-excellent inter-model agreement in risk ranking. This supports the broader concept that radiation-induced cancer risk estimates depend strongly on the assumptions, endpoints, and populations underlying each model [22].

More recent approaches include ensemble modeling, in which alternative sub-models for radiation quality, dose, dose-rate effectiveness factor (DDREF), excess risk transfer, latency, and tissue-specific risk are combined within a unified probabilistic framework [8]. Instead of relying on a single set of fixed assumptions, ensemble models assign probability distributions or weights to competing model components and repeatedly sample these distributions to generate a range of possible cancer risk estimates [8]. Uncertainty propagation is performed by carrying forward the variability associated with each model component, such as radiation quality factors, DDREF assumptions, epidemiological transfer parameters, and latency functions, into the final risk distribution [8]. This approach allows the model output to be expressed not only as a central estimate but also as confidence intervals or upper-percentile values, particularly the upper 95% confidence level used for radiation protection decisions. These methods better capture the uncertainty in the range that is most relevant to mission duration limits, astronaut selection, and operational risk management [8].

An important methodological distinction is that earlier models mainly estimated central risk values, whereas more recent frameworks increasingly emphasize uncertainty propagation and upper confidence limits, which are more relevant for astronaut selection, mission approval, and operational radiation protection planning. From an imaging-oriented perspective, these risk models also provide a conceptual framework for the future integration of quantitative biomarkers derived from CT, MRI, PET, and other tomographic modalities. Although such approaches are not yet part of the operational astronaut cancer risk assessment, they may become relevant for monitoring radiation-related tissue changes, supporting early detection strategies, and improving individualized longitudinal risk stratification.

## 4. Potential Role of Tomographic Imaging in Astronaut Radiation Risk Assessment

Although current astronaut cancer risk models are primarily based on dosimetry, radiobiology, and epidemiological risk transfer, tomographic imaging may provide complementary information that is not captured by these models. Currently, CT, MRI, PET, and radiomics-derived biomarkers are not validated components of operational astronaut cancer risk assessment. However, they may contribute to future individualized monitoring by detecting organ-specific tissue changes before, during, and after long-duration missions.

The realistic role of tomographic imaging is longitudinal health monitoring rather than the direct replacement of radiation risk models. MRI has been used in space medicine to document structural brain changes after spaceflight, including alterations in brain position, cerebrospinal fluid spaces, ventricular volume, and gray matter distribution [23,24,25]. Although these findings are largely related to microgravity and fluid redistribution rather than radiation carcinogenesis, they demonstrate that tomographic imaging can detect measurable spaceflight-associated tissue changes [23,24,25]. This supports the broader concept that imaging can be used as a longitudinal tool for monitoring radiation-sensitive organs during exploration missions.

Several potential imaging endpoints are relevant to space radiation research. CNS imaging with MRI can be used to assess white matter integrity, brain volume changes, vascular alterations, and possible neuroinflammatory or neurodegenerative effects [23,24,25]. Bone marrow imaging may help evaluate the hematopoietic tissue composition, marrow fat fraction, and radiation-related marrow injury [26]. Lung CT could be relevant for detecting inflammatory or fibrotic changes after radiation exposure, particularly in the context of solar particle events or mixed-field irradiation [26]. Vascular imaging may help characterize endothelial dysfunction, accelerated vascular aging, and radiation-associated cardiovascular risk [26]. PET imaging can provide functional information on inflammation, metabolism, hypoxia, and tissue repair processes, depending on tracer availability and mission constraints [26].

In oncology-oriented surveillance, tomographic imaging may also contribute to the early detection and follow-up of radiation-associated malignancies, although no specific CT, MRI, PET, or radiomics biomarkers have yet been validated to predict cancer development in astronauts. Radiomics-based tissue phenotyping could be particularly useful in future research because it allows the extraction of quantitative features from serial imaging datasets, potentially capturing subtle structural or textural changes that are not visible on conventional visual interpretation [26]. Such features could eventually be integrated with dosimetry, molecular biomarkers, demographic variables and mission-specific exposure profiles.

Recent advances in space medicine suggest that the oral cavity may represent a uniquely accessible biological observatory for investigating the long-term consequences of deep-space radiation exposure and its potential relationship with cancer development. The oral cavity is particularly attractive for astronaut health surveillance because it can be examined repeatedly using non-invasive, low-resource procedures that are compatible with the operational constraints of long-duration space missions. In addition, saliva collection and oral imaging can be performed serially with minimal crew burden, enabling longitudinal monitoring of biological responses to space radiation and other environmental stressors without the need for invasive sampling. While current astronaut cancer risk models primarily rely on epidemiological extrapolations from terrestrial radiation cohorts and experimental radiobiological data, significant uncertainties remain regarding the biological effects of high-linear energy transfer (high-LET) particles characteristic of galactic cosmic rays (GCRs), particularly when combined with chronic microgravity, circadian disruption, and psychosocial stressors [27,28].

The oral cavity may represent a relevant and accessible compartment for astronaut radiation monitoring because oral mucosa, salivary glands, periodontal tissues, and alveolar bone are biologically dynamic and potentially sensitive to radiation-related oxidative stress, inflammation, DNA damage, and immune changes. Salivary biomarkers may offer a non-invasive approach for longitudinal assessment, while oral imaging techniques, including low-dose CBCT, MRI, and AI-based radiomics, could help detect subtle structural or tissue changes. However, these applications remain exploratory and should be considered complementary to established dosimetry- and epidemiology-based risk models [27,28].

The main limitation of this study is that direct astronaut evidence remains limited. Most current data supporting imaging-based assessments of radiation injury come from terrestrial radiation oncology, radiobiology, and experimental models rather than from astronauts exposed to deep-space radiation [26]. The role of tomographic imaging in this field should be interpreted as both emerging and complementary. Future studies should aim to integrate standardized pre- and post-flight imaging, radiation dosimetry, biological markers of DNA damage and inflammation, and long-term clinical follow-up. This would help determine whether imaging-derived biomarkers can improve individualized risk stratification and make astronaut cancer risk models more relevant to the imaging and tomography communities.

## 5. Literature Search Strategy

This narrative review was based on a structured, targeted literature search aimed at identifying the most relevant quantitative models, methodological studies, and technical reports on cancer risk estimation in astronauts exposed to space radiation. The search was performed on 15 May 2026 and covered publications from database inception to 15 May 2026. The primary search was conducted in PubMed using the following strategy: (“space radiation”[Title/Abstract] OR “cosmic radiation”[Title/Abstract] OR “galactic cosmic rays”[Title/Abstract]) AND (astronaut*[Title/Abstract] OR “space mission”[Title/Abstract] OR spaceflight[Title/Abstract] OR “deep space”[Title/Abstract]) AND (“cancer risk estimation”[Title/Abstract] OR “cancer risk projection”[Title/Abstract] OR “cancer risk assessment”[Title/Abstract] OR “radiation risk model”[Title/Abstract] OR REID[Title/Abstract]). To broaden the search beyond the PubMed-indexed biomedical literature, additional searches were performed in Scopus and Web of Science using comparable keyword combinations. Institutional and technical sources were also screened, including the NASA Technical Reports Server, National Academies reports, NASA Space Cancer Risk documentation, and relevant NASA Radiation Protection Standards. Additional studies were identified by manually screening the reference lists of the selected articles and reports.

The PubMed search retrieved 59 records. After removal of one duplicate/preprint record, 58 records were screened by title and abstract for direct relevance to quantitative astronaut cancer-risk projection. Thirty-nine records were excluded at this stage because they focused on animal or cellular biology only, non-cancer endpoints, dosimetry without cancer-risk modeling, or broad reviews/commentaries. Nineteen full-text articles or reports were then assessed for eligibility as quantitative or methodological cancer-risk studies. Ten full-text sources were excluded because they addressed background radiobiology or biomarkers without model output, operational dosimetry or countermeasures only, or non-quantitative narrative topics. The remaining 9 major quantitative or methodological studies/reports were included in the qualitative synthesis and summarized in Table 1.

Studies and reports were included if they addressed quantitative cancer risk estimation, cancer risk projection, REID-based assessment, uncertainty modeling, radiation quality factors, DDREF, ensemble modeling, NASA radiation exposure standards, or methodological limitations of astronaut radiation risk assessment. Original studies, methodological reports, technical evaluations, and institutional reports were included in the review. Broad narrative reviews, editorials, commentaries, and papers not directly addressing quantitative cancer risk estimation were excluded, although selected reviews were used when they provided relevant background on space radiation biology, radiological protection, or tomographic imaging.

Because this article was designed as a narrative, non-systematic review, formal systematic review procedures, including protocol registration, duplicate independent screening, full PRISMA-based reporting, and structured risk-of-bias assessment, were not applied. However, to improve transparency, the selection process was documented using a simplified flow diagram. The final qualitative synthesis focused on the 9 selected major quantitative or methodological studies/reports, which were chosen because they introduced or substantially refined astronaut cancer risk models, provided operational REID-based estimates, addressed uncertainty propagation, evaluated NASA radiation standards, or discussed biological and translational limitations relevant to long-duration exploration missions.

**Figure 1:**
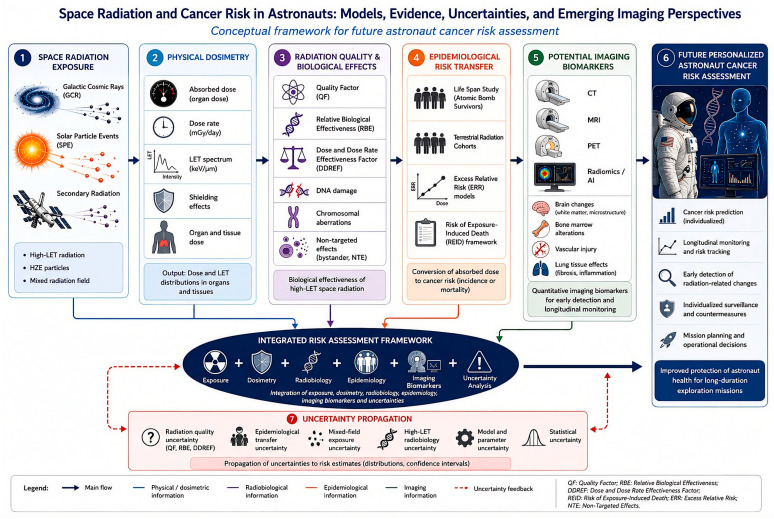
Conceptual framework for future astronaut cancer risk assessment. The diagram summarizes the integration of space radiation exposure, physical dosimetry, radiobiological modifiers, epidemiological risk models, imaging biomarkers, and uncertainty propagation to support personalized cancer risk assessment and long-term astronaut health monitoring.

**Figure 2:**
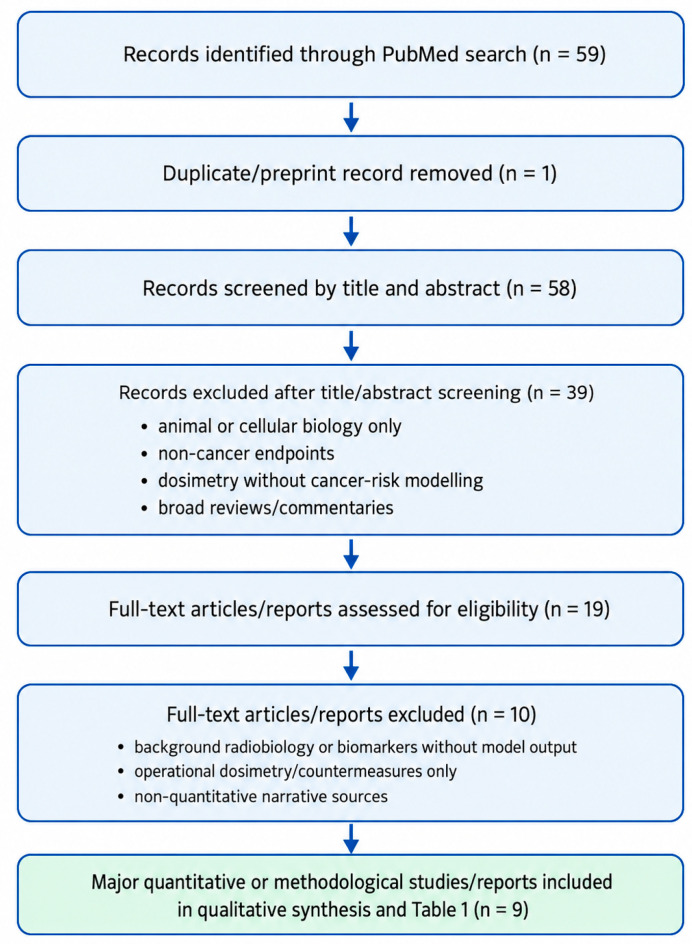
Flow diagram of the literature selection process. The diagram summarizes the targeted PubMed search and study-selection workflow used for this narrative review. Fifty-nine records were initially identified; after removal of one duplicate/preprint record, 58 records were screened by title and abstract. Thirty-nine records were excluded at this stage, and 19 full-text articles or reports were assessed for eligibility. After excluding 10 full-text sources that did not provide quantitative or methodological cancer-risk model outputs, 9 major studies/reports were included in the qualitative synthesis and summarized in Table 1.

During the preparation of this manuscript, the authors used ChatGPT-5.6 Thinking, accessed in July 2026, to assist with grammar correction and with the generation and refinement of graphical elements for Figure 1 and Figure 2.

A flow diagram of the study selection process is provided in Figure 2. These studies and reports are summarized in Table 1.

## 6. Key Quantitative Findings from Major Studies

The main quantitative studies included in this narrative review are summarized in Table 1, which compares the model type, radiation scenario, endpoint, uncertainty treatment, and operational relevance across the selected studies. To increase the analytical value of the comparison, the table reports not only the study type and main quantitative findings, but also the mission or exposure scenario, dominant source of uncertainty, and biological or operational endpoint used. This structure highlights how the selected models differ in terms of assumptions, uncertainty treatment, biological endpoints, and operational relevance for astronaut radiation protection.

Particular attention should be given to the series of studies by Cucinotta and colleagues, which progressively refined astronaut cancer risk estimation from mission-level uncertainty analysis to operational REID-based projections, uncertainty distribution modeling, non-targeted effect scenarios, and updated NSCR formulations incorporating revised QF and DDREF parameter distributions.

### 6.1. Quantitative Cancer Risk Projection and Operational Mission Limits

Quantitative cancer risk projection in astronauts has evolved from early particle-based estimates to operational frameworks designed to inform mission duration limits and radiation protection standards. Initial modeling efforts focused on translating the physical characteristics of galactic cosmic rays into projected biological risks. In this context, Curtis et al. provided one of the earliest quantitative estimates of excess cancer mortality, reporting that under 10 g/cm^2^ aluminum shielding at solar minimum, the projected 1-year excess cancer mortality was 1.3% in women and 1.1% in men, with uncertainty spanning approximately 4-fold to 15-fold [21]. These findings established a quantitative basis for astronaut risk assessment, while also showing that uncertainty was a dominant feature of the problem [21].

A further advance was made by Peterson et al. [7], who applied a Monte Carlo mixture model to estimate lifetime non-leukemia cancer incidence after radiation exposure. For an exposure expressed as 1 Sv dose equivalent/effective dose, projected risks in men were 2.77% at age 45 and 2.20% at age 55, whereas in women they were 2.98% at age 45 and 2.44% at age 55 [7]. The associated 90% confidence intervals were wide, extending up to 11.34% in men and 11.70% in women, thereby underscoring the uncertainty inherent in the risk transfer from terrestrial epidemiological datasets. The same study also demonstrated the strong dependence of projected cancer risk on particle type and LET. Whereas 200 MeV protons were associated with a lifetime risk of only 2.68 × 10^−2^%, the estimated risks increased to 14.2% for 1 MeV alpha particles and 23.7% for 600 MeV iron ions, highlighting the disproportionate contribution of high-LET particles to the space radiation risk [7].

More recent studies have shifted attention from lifetime cancer incidence alone to operationally relevant endpoints, particularly the risk of exposure-induced death (REID). In the earlier NASA framework, permissible career exposure limits were based on sex- and age-specific estimates of cancer mortality risk, with the goal of not exceeding a 3% REID threshold at the upper 95% confidence level (CL) [30]. Within that historical modeling framework, Cucinotta et al. estimated that a 1-year ISS mission would generally remain within the limit for typical crew demographics, whereas under solar minimum conditions, the upper 95% confidence bound could exceed the 3% REID threshold after approximately 18 months in women and 24 months in men. These values should be interpreted as historical model-based projections, not as current NASA operational dose limits. The NASA exposure standard has moved from sex- and age-specific REID-based career limits to a universal career effective dose limit of 600 mSv for spaceflight radiation exposure, applied regardless of sex or age [11]. This standard was introduced to provide a single operational limit while remaining anchored to the principles of cancer risk protection. Accordingly, older REID-based estimates remain useful for understanding the evolution of astronaut cancer risk modeling and uncertainty, but they should not be presented as the current NASA exposure standards [10,11].

In the same analysis, the estimated probability of causation exceeded 50% for several cancers in astronauts completing two or more ISS missions or accumulating 18 months or more in orbit [30].

The difficulty in maintaining acceptable risk becomes even more evident in exploration-class missions. In a Mars mission analysis, Cucinotta et al. estimated that the projected cancer risk was associated with uncertainty on the order of 400–600%, with uncertainty in radiation quality factors representing the largest single contributor [29]. Their findings suggested that missions lasting more than 90 days beyond Earth’s magnetic field would not satisfy the existing low-Earth-orbit radiation protection standards when uncertainty bounds were included [29]. This operational perspective was subsequently evaluated by the National Research Council, which confirmed the centrality of REID in NASA’s updated framework while also noting the model’s substantial complexity and the limited transparency of several parameter assumptions [10,11].

More recently, Stegeman et al. reaffirmed that the NASA Space Cancer Risk model, which has been used operationally since 2013, remains fundamentally anchored to the Life Span Study of atomic bomb survivors as its core epidemiological basis [5]. Overall, these studies show that quantitative astronaut cancer risk assessment has become increasingly sophisticated and operationally relevant, but remains strongly conditioned by sex, age, mission duration, radiation quality, and the assumptions used to extrapolate terrestrial cancer risk data to deep-space environments.

### 6.2. Uncertainty, Model Refinement, and Emerging Biological Modifiers

A second major line of research has focused on the structure of uncertainty in astronaut cancer risk estimation and the biological factors that may modify the projected risk beyond conventional assumptions. In this area, the contribution of Cucinotta et al. has been particularly influential, because it shifted attention from central risk estimates alone to the broader problem of how uncertainty is generated, propagated, and interpreted in mission planning.

In one of the earlier analyses of exploration-class missions, Cucinotta et al. showed that projected cancer risk for Mars scenarios was associated with uncertainty of approximately 400–600%, a range large enough to challenge the practical usefulness of any single numerical estimate [29]. Their analysis indicated that the dominant source of uncertainty was the radiation quality factor, namely the parameter used to translate physical dose into biologically effective dose for different radiation types [20]. This finding was especially relevant for galactic cosmic ray exposure, where high-charge and high-energy (HZE) particles contribute disproportionately to biologic damage despite representing only a small fraction of the particle field. The same study further suggested that missions lasting more than 90 days beyond Earth’s magnetic field would fail to satisfy existing low-Earth-orbit radiation protection standards once uncertainty bounds were considered [29].

Subsequent work extended this perspective by examining not only the magnitude, but also the shape of uncertainty distributions. Cucinotta et al. argued that the acceptability of uncertainty should not be judged solely by its width, but also by the characteristics of the underlying probability distribution function (PDF) [31]. Within NASA’s operational framework, which sets an acceptable limit of no more than 3% probability of cancer fatality at the 95% confidence level, the authors proposed that a distribution with only modest skewness may be more acceptable than one with a long upper tail, even if the nominal uncertainty is similar. Using this approach, they estimated PDFs and the number of mission “safe days” for several exposure scenarios, defined as the mission duration below which the accepted risk limit would not be exceeded. This work also highlighted additional contributors to uncertainty, including possible non-cancer late effects, differences in tumor spectrum and lethality observed in animal studies after HZE exposure, and the potential role of non-targeted effects (NTEs) in amplifying low-dose responses [31].

An important methodological advance was represented by later revisions of the NASA Space Cancer Risk (NSCR) model, in which Cucinotta et al. updated the PDFs for two key parameters: the quality factor (QF) and the dose and dose-rate reduction effectiveness factor (DDREF). These revisions incorporated heavy-ion tumor data from five tissues—liver, colorectal, intestinal, lung, and Harderian gland—as well as results from fission neutron experiments. Unlike earlier formulations, the revised model treated leukemia, solid cancers, and liver cancer separately, in part because mouse studies suggested that liver tumors may show higher relative biological effectiveness (RBE) than other tumor types. The model was then applied to three mission scenarios: a 1-year deep-space mission, a 1-year ISS mission, and a 940-day Mars mission. In all three cases, NASA exposure limits were found to be approached or exceeded, while the remaining overall model uncertainty was still on the order of 3-fold [20]. The authors concluded that reducing this uncertainty to 2-fold or less would require substantially more extensive animal carcinogenesis studies capable of addressing tissue, sex, and genetic variability [20].

Beyond parameter refinement, recent studies have questioned whether conventional targeted-effect models may systematically underestimate exploration risk. In this regard, Cucinotta and Cacao proposed that non-targeted effects could significantly amplify fatal cancer risk during galactic cosmic ray exposure [13]. In their analysis, incorporating NTEs increased projected fatal cancer risk by approximately 2- to 4-fold in a 365-day lunar mission and a 500-day Phobos mission, suggesting that biological responses in non-irradiated bystander cells may substantially alter the expected dose–response relationship [13].

The implications of this broader uncertainty landscape were further reinforced by Simonsen et al., who developed an ensemble modeling framework integrating multiple sub-models for radiation quality, DDREF, excess risk, and latency [12]. Their results showed that ensemble projections were broadly consistent with NASA’s standard model at the median ± 1 SD, but diverged substantially at the upper 95% confidence level, precisely the range most relevant to mission approval and operational decision-making [10]. Taken together, these studies indicate that the major challenge in astronaut cancer risk assessment is not only estimating a central value but understanding the biological and model-based processes that widen uncertainty at exploration-relevant doses.

## 7. Sources of Uncertainty and Model Limitations

Cancer risk estimation in astronauts remains affected by substantial uncertainty at multiple levels, including radiation physics, radiobiology, epidemiologic transfer, and statistical modeling. Early particle-based and transfer models already showed this limitation, with broad uncertainty intervals around both excess cancer mortality and lifetime non-leukemia cancer incidence estimates, as summarized in Section 6.1 and Table 1.

A major limitation is that current NASA models still depend heavily on the Life Span Study of atomic bomb survivors, whose acute low-LET exposures differ substantially from chronic mixed-field high-LET space radiation. Additional uncertainty arises from sex- and age-dependent transfer functions, DDREF assumptions, shielding transport, and quality factors for HZE particles. Notably, incorporation of non-targeted effects increased predicted fatal cancer risks by 2- to 4-fold in 365-day to 500-day exploration scenarios, underscoring how sensitive current models remain to limited radiobiological data and model assumptions [20].

The transfer of risk estimates from the Hiroshima/Nagasaki Life Span Study cohorts to astronaut populations remains one of the central methodological limitations of current models. The Life Span Study is based primarily on acute, relatively high-dose-rate, low-LET photon and neutron exposure, whereas astronauts are exposed to chronic, low-dose-rate, mixed radiation fields that include protons, alpha particles, and high-charge and high-energy nuclei [32]. These differences affect dose-rate effectiveness, radiation quality, biological repair kinetics, and the shape of the dose–response relationship. In addition, the astronaut population differs substantially from the general exposed populations in the Life Span Study because astronauts are highly selected, medically screened, and generally healthier than the average population. Risk transfer requires assumptions about sex, age at exposure, baseline cancer rates, latency, competing risks, and organ-specific sensitivity, all of which contribute to uncertainty in projected cancer risk [33].

Animal models provide essential mechanistic information on high-LET radiation effects, tissue-specific carcinogenesis, genomic instability, and non-targeted effects, but their translation to human astronaut risk is also limited. Rodent and other experimental models differ from humans in lifespan, tumor spectrum, immune response, DNA repair capacity, genetic background, and radiation sensitivity [34]. Experimental irradiation conditions may also differ from operational spaceflight exposure, particularly with respect to dose rate, fractionation, particle composition, and mixed-field complexity. As a result, animal studies are valuable for identifying biological mechanisms and refining model parameters, but they cannot be used as direct substitutes for human epidemiological data. These translational limitations should be considered when interpreting model projections for long-duration lunar or Mars missions [35].

### Radiological Protection Considerations Relevant to Cancer-Risk Modeling

From a radiological protection perspective, astronaut cancer-risk estimates should be interpreted as decision-support quantities rather than as stand-alone predictions. Space radiation protection is founded on justification, limitation, and optimization; in this setting, effective dose provides a practical common metric for exposure limitation, but it cannot fully describe individual risk in a mixed field dominated by protons, alpha particles, HZE ions, secondary neutrons, and temporally variable solar particle events [18]. Model outputs such as REID, lifetime cancer incidence, and upper-confidence estimates should be read together with organ dose, dose equivalent, LET spectra, radiation quality assumptions, and uncertainty propagation [36].

Operationally, the radioprotection problem differs for GCR and SPE exposure. GCR exposure is chronic, highly penetrating, and only partly reducible by passive shielding because nuclear interactions in spacecraft materials and tissue can generate secondary radiation; conversely, SPE exposure is episodic and more amenable to real-time monitoring, sheltering, mission-timing constraints, and optimized local shielding [37]. Hydrogen-rich and composite materials can improve shielding performance relative to conventional aluminum in specific scenarios, but shielding design must consider mass constraints, secondary particle production, and the distinction between acute-effect prevention and late stochastic cancer-risk reduction [38]. For this reason, a realistic astronaut cancer-risk framework should retain the original dosimetric and epidemiological model structure while explicitly linking it to radiological protection practices, including as low as reasonably achievable (ALARA)-based planning, environmental and personal dosimetry, event-specific dose reconstruction, and transparent communication of uncertainty [30].

## 8. Future Directions

Future progress in astronaut cancer risk estimation will depend not only on reducing the gap between terrestrial epidemiology and the biological complexity of deep-space radiation exposure, but also on exploring how quantitative imaging approaches may complement current dosimetric and radiobiological models. Potential developments include CT-, MRI-, and PET-based assessment of radiation-related tissue changes, imaging biomarkers of early organ injury, and radiomics-based phenotyping for longitudinal, individualized monitoring.

Although imaging biomarkers are not yet incorporated into operational astronaut cancer risk models, several existing lines of evidence support their future relevance. Longitudinal MRI studies in astronauts have demonstrated measurable structural brain changes after spaceflight, including alterations in brain position, cerebrospinal fluid spaces, gray matter distribution, and ventricular volume [23,24,25].

These studies do not directly measure radiation-induced cancer risk, but they show that tomographic imaging can detect spaceflight-associated tissue and fluid changes in humans over time. In parallel, radiation oncology and radiobiology studies have shown that quantitative MRI, PET, SPECT, and CT can detect radiation-induced normal tissue injury, including inflammatory, vascular, fibrotic, and metabolic changes before or alongside clinical manifestations [26]. These approaches provide a methodological foundation for applying imaging-based monitoring to space radiation research. For future lunar and Mars missions, the most realistic role of CT, MRI, PET, and radiomics is therefore not to replace dosimetric or epidemiological risk models, but to complement them by providing longitudinal, organ-specific information on tissue response. Potential applications include MRI-based assessment of central nervous system changes, PET-based evaluation of inflammatory or metabolic tissue responses, CT-based assessment of radiation-related structural injury, and radiomics-based extraction of quantitative features from serial imaging datasets [26]. However, these approaches should currently be regarded as exploratory, because no imaging biomarker has yet been validated as a direct predictor of radiation-induced cancer risk in astronauts. Future studies should therefore integrate imaging endpoints with dosimetry, molecular biomarkers, organ-specific functional measures, and long-term clinical follow-up.

A major priority is refinement of quality factors, DDREF, and sex- and age-specific transfer functions, because current projections remain strongly affected by broad upper confidence limits [5].

Wider use of ensemble frameworks may improve uncertainty communication, especially at the upper 95% confidence level, which determines operational mission constraints. Personalized modeling should also be expanded by integrating individual variables such as sex, age, smoking history, and potentially genomic susceptibility markers [12]. In parallel, improved shielding optimization, biologically informed transport models, and better characterization of non-targeted effects, which may increase fatal cancer predictions by 2- to 4-fold, will be essential for future lunar and Mars mission planning. In this perspective, the next major advance in astronaut cancer risk estimation will likely come not from a single new coefficient or dataset, but from integrated frameworks combining space-relevant radiobiology, personalized susceptibility factors, uncertainty propagation, and mission-specific dosimetry.

## 9. Conclusions

Early particle-based and transfer models provided quantitative estimates of astronaut cancer risk but also showed substantial uncertainty.

Cancer risk estimation remains a central limitation for long-duration human exploration beyond low Earth orbit. Current models have progressed from deterministic particle-fluence approaches to REID-based, probabilistic, and ensemble frameworks, but their operational use remains constrained by substantial uncertainty in radiation quality factors, DDREF assumptions, epidemiological risk transfer, dose-rate effects, and non-targeted biological responses [18]. The main priority for future research is therefore not simply to generate additional point estimates of cancer risk, but to improve the reliability, comparability, and biological validity of risk prediction frameworks. First, existing models should be harmonized and validated against space-relevant radiobiological data, including high-LET heavy-ion exposure, chronic low-dose-rate irradiation, mixed-field conditions, and tissue-specific carcinogenesis endpoints. Second, uncertainty propagation should be reported transparently, particularly at upper confidence limits that directly influence mission-duration constraints and astronaut selection. Third, quantitative imaging biomarkers derived from CT, MRI, PET, and radiomics should be investigated cautiously as exploratory complementary tools for longitudinal monitoring of radiation-related tissue effects, while recognizing that they are not yet validated components of operational astronaut cancer risk models. Ultimately, future astronaut cancer risk assessment will require integrated, individualized frameworks combining mission-specific dosimetry, radiobiological evidence, epidemiological risk transfer, genetic and demographic susceptibility factors, and imaging-based tissue monitoring. Such approaches may support more transparent risk communication, more personalized surveillance strategies, and safer planning of lunar and Mars exploration missions.

## Figures and Tables

**Table 1:** Main quantitative studies on cancer risk estimation in astronauts exposed to space radiation.

First Author (Year)	Model Type	Radiation Scenario	Endpoint	Key Quantitative Findings	Uncertainty Treatment	Operational Relevance
**Curtis (1995) [21]**	Risk cross sections per particle fluence	GCR exposure under 10 g/cm^2^ aluminum shielding at solar minimum	Excess cancer mortality	Under 10 g/cm^2^ Al shielding at solar minimum, 1-year excess cancer mortality was 1.3% in women and 1.1% in men, with uncertainty ranging from 4-fold to 15-fold.	Radiation quality, particle fluence-to-risk conversion, and shielding assumptions	Early deterministic model with limited biological and epidemiological refinement
**Peterson (1999) [7]**	Monte Carlo mixture model	1 Sv exposure; age- and sex-specific shuttle/ISS-related scenarios	Lifetime non-leukemia cancer incidence	After 1 Sv, the projected non-leukemic lifetime cancer incidence was 2.77% in men aged 45, 2.20% in men aged 55, 2.98% in women aged 45, and 2.44% in women aged 55; the upper 90% confidence limits exceeded 11%.	Risk transfer from terrestrial cohorts, sex and age dependence, particle type, and LET	Limited direct validation in astronauts and dependence on terrestrial epidemiological extrapolation
**National Research Council (2012) [20]**	Technical evaluation of NASA risk model	NASA astronaut radiation protection framework for exploration missions	REID framework and uncertainty	The REID was confirmed as NASA’s operational endpoint, and key uncertainties related to model assumptions, radiation quality factors, and transport of GCR and SPE exposures were emphasized.	Model structure, QF assumptions, GCR/SPE transport, and epidemiological transfer	Technical evaluation rather than a primary quantitative risk projection study
**Cucinotta et al. (2001) [29]**	Mars mission risk projection and uncertainty analysis	Mars exploration scenarios beyond Earth’s magnetic field	Cancer mortality risk uncertainty	For Mars mission scenarios, the projected cancer risk uncertainty was approximately 400–600%; missions longer than 90 days beyond Earth’s magnetic field were unlikely to satisfy low-Earth-orbit standards once uncertainty bounds were included.	Radiation quality factors, high-LET biological effectiveness, and dose-rate assumptions	Very large uncertainty range limits operational interpretability
**Cucinotta (2014) [30]**	Mission-specific risk projection for multiple ISS missions	1-year and repeated ISS missions, especially near solar minimum	REID and probability of causation	NASA’s 3% REID threshold may be exceeded after approximately 18 months in women and 24 months in men near the solar minimum; the probability of causation exceeded 50% for several cancers after prolonged or repeated ISS missions.	Sex, age, mission duration, solar cycle, and radiation quality assumptions	Mainly focused on ISS/low-Earth-orbit exposure rather than deep-space missions
**Cucinotta et al. (2015) [31]**	Probability distribution/“safe days” framework	Mission-duration scenarios constrained by NASA’s 3% fatal cancer risk limit	Fatal cancer risk and mission-duration limits	Estimated mission “safe days” under NASA’s 3% fatal cancer risk limit at the 95% confidence level; highlighted the role of skewed uncertainty distributions and high-LET assumptions in limiting allowable mission duration.	Shape of uncertainty distributions, high-LET assumptions, and upper-tail risk	Strong dependence on assumed probability distribution functions
**Cucinotta and Cacao (2017) [13]**	Non-targeted effects model	365-day lunar and 500-day Phobos mission scenarios	Fatal cancer risk in exploration missions	Incorporating non-targeted effects increased the projected fatal cancer risk by approximately 2- to 4-fold in the 365-day lunar and 500-day Phobos mission scenarios.	Biological assumptions regarding non-targeted effects and bystander responses	Limited direct human validation of NTE-based cancer risk projections
**Simonsen et al. (2021) [12]**	Ensemble risk model	Space radiation cancer risk projections using multiple sub-model combinations	Cancer risk projection uncertainty	The ensemble estimates were broadly consistent with NASA’s standard model around the median but diverged substantially at the upper 95% confidence level.	Radiation quality, DDREF, excess risk transfer, latency, and model weighting	Results depend on the selected sub-models and weighting strategy
**Stegeman et al. (2025) [5]**	NSCR methodological update	NASA astronaut cancer risk assessment framework	Cancer risk assessment framework	The NASA Space Cancer Risk model remains strongly anchored to the Life Span Study as its epidemiological basis.	Transfer from acute terrestrial low-LET exposure to chronic mixed-field space radiation	Continued dependence on Life Span Study data and limited direct astronaut cancer outcome data

## Data Availability

No new datasets were generated for this narrative review. The quantitative data summarized in Table 1 were obtained from the cited published sources.

## Abbreviations

**CT**: computed tomography; **DDREF**: dose and dose-rate effectiveness factor; **GCR**: galactic cosmic rays; **HZE**: high-charge and high-energy particles; **ISS**: International Space Station; **LET**: linear energy transfer; **MRI**: magnetic resonance imaging; **NASA**: National Aeronautics and Space Administration; **NSCR**: NASA Space Cancer Risk model; **NTE**: non-targeted effects; **PDF**: probability distribution function; **PET**: positron emission tomography; **QF**: quality factor; **RBE**: relative biological effectiveness; **REID**: risk of exposure-induced death; **SPE**: solar particle event.

## References

[1] Fogtman A., Baatout S., Baselet B., Berger T., Hellweg C.E., Jiggens P., La Tessa C., Narici L., Nieminen P., Sabatier L. (2023). Towards sustainable human space exploration-priorities for radiation research to quantify and mitigate radiation risks. npj Microgravity.

[2] Montesinos C.A., Khalid R., Cristea O., Greenberger J.S., Epperly M.W., Lemon J.A., Boreham D.R., Popov D., Gorthi G., Ramkumar N. (2021). Space Radiation Protection Countermeasures in Microgravity and Planetary Exploration. Life.

[3] Guo Z., Zhou G., Hu W. (2022). Carcinogenesis induced by space radiation: A systematic review. Neoplasia.

[4] Shavers M., Semones E., Tomi L., Chen J., Straube U., Komiyama T., Shurshakov V., Li C., Rühm W. (2024). Space agency-specific standards for crew dose and risk assessment of ionising radiation exposures for the International Space Station. Z. Med. Phys..

[5] Stegeman L., Slaba T.C., Huff J.L., Semones E., Zawaski J.A., Saha J. (2025). Utilizing the Life Span Study data in NASA astronaut cancer risk assessment. Carcinogenesis.

[6] Barcellos-Hoff M.H., Blakely E.A., Burma S., Fornace A.J., Gerson S., Hlatky L., Kirsch D.G., Luderer U., Shay J., Wang Y. (2015). Concepts and challenges in cancer risk prediction for the space radiation environment. Life Sci. Space Res..

[7] Peterson L.E., Cucinotta F.A. (1999). Monte Carlo mixture model of lifetime cancer incidence risk from radiation exposure on shuttle and international space station. Mutat. Res./Fundam. Mol. Mech. Mutagen..

[8] Sridharan D.M., Asaithamby A., Blattnig S.R., Costes S.V., Doetsch P.W., Dynan W.S., Hahnfeldt P., Hlatky L., Kidane Y., Kronenberg A. (2016). Evaluating biomarkers to model cancer risk post cosmic ray exposure. Life Sci. Space Res..

[9] Cucinotta F.A., Kim M.-H.Y., Chappell L.J. (2013). Space Radiation Cancer Risk Projections and Uncertainties—2012.

[10] NASA Office of the Chief Health and Medical Officer (2023). OCHMO-TB-020: Design for Ionizing Radiation Protection.

[11] (2023). Spaceflight Human-System Standard, Volume 1: Crew Health.

[12] Simonsen L.C., Slaba T.C. (2021). Improving astronaut cancer risk assessment from space radiation with an ensemble model framework. Life Sci. Space Res..

[13] Cucinotta F.A., Cacao E. (2017). Non-Targeted Effects Models Predict Significantly Higher Mars Mission Cancer Risk than Targeted Effects Models. Sci. Rep..

[14] Walsh L., Hafner L., Berger T., Matthiä D., Schneider U., Straube U. (2024). European astronaut radiation related cancer risk assessment using dosimetric calculations of organ dose equivalents. Z. Med. Phys..

[15] Norbury J.W., Schimmerling W., Slaba T.C., Azzam E.I., Badavi F.F., Baiocco G., Benton E., Bindi V., Blakely E.A., Blattnig S.R. (2016). Galactic cosmic ray simulation at the NASA Space Radiation Laboratory. Life Sci. Space Res..

[16] Kennedy A.R. (2014). Biological effects of space radiation and development of effective countermeasures. Life Sci. Space Res..

[17] Papadopoulos A., Kyriakou I., Incerti S., Santin G., Nieminen P., Daglis I.A., Li W., Emfietzoglou D. (2023). Space radiation quality factor for Galactic Cosmic Rays and typical space mission scenarios using a microdosimetric approach. Radiat. Environ. Biophys..

[18] Chancellor J.C., Blue R.S., Cengel K.A., Auñón-Chancellor S.M., Rubins K.H., Katzgraber H.G., Kennedy A.R. (2018). Limitations in predicting the space radiation health risk for exploration astronauts. npj Microgravity.

[19] Durante M., Cucinotta F.A. (2011). Physical basis of radiation protection in space travel. Rev. Mod. Phys..

[20] National Research Council, Division on Engineering, Physical Sciences, Space Studies Board, Committee for Evaluation of Space Radiation Cancer Risk Model (2012). Technical Evaluation of the NASA Model for Cancer Risk to Astronauts Due to Space Radiation.

[21] Curtis S.B., Nealy J.E., Wilson J.W. (1995). Risk cross sections and their application to risk estimation in the galactic cosmic-ray environment. Radiat. Res..

[22] Quaia E., Zanon C., Torchio R., Dughiero F., De Monte F., Paiusco M. (2025). Variability Between Radiation-Induced Cancer Risk Models in Estimating Oncogenic Risk in Intensive Care Unit Patients. Tomography.

[23] Roberts D.R., Albrecht M.H., Collins H.R., Asemani D., Chatterjee A.R., Spampinato M.V., Zhu X., Chimowitz M.I., Antonucci M.U. (2017). Effects of Spaceflight on Astronaut Brain Structure as Indicated on MRI. N. Engl. J. Med..

[24] Koppelmans V., Bloomberg J.J., Mulavara A.P., Seidler R.D. (2016). Brain structural plasticity with spaceflight. npj Microgravity.

[25] Hupfeld K.E., McGregor H.R., Lee J.K., E Beltran N., Kofman I.S., E De Dios Y., A Reuter-Lorenz P., Riascos R.F., Pasternak O., Wood S.J. (2020). The Impact of 6 and 12 Months in Space on Human Brain Structure and Intracranial Fluid Shifts. Cereb. Cortex Commun..

[26] Robbins M.E., Brunso-Bechtold J.K., Peiffer A.M., Tsien C.I., Bailey J.E., Marks L.B. (2012). Imaging Radiation-Induced Normal Tissue Injury. Radiat. Res..

[27] Zanon C., Chiaravalloti A., Crimì F., Favero V., Santarelli F., Quaia E., Bollero P., Belfiore M.P., Basilicata M. (2026). Cancer Risk Estimation and Radiation-Protective Shielding in Dental Cone-Beam Computed Tomography: An Updated Narrative Review. Appl. Sci..

[28] Khijmatgar S., Pellegrini M., Ghizzoni M., Del Fabbro M. (2025). Effect of Microgravity and Space Radiation Exposure on Human Oral Health: A Systematic Review. Biophysica.

[29] Cucinotta F.A., Schimmerling W., Wilson J.W., Peterson L.E., Badhwar G.D., Saganti P.B., Dicello J.F. (2001). Space radiation cancer risks and uncertainties for Mars missions. Radiat. Res..

[30] Cucinotta F.A. (2014). Space radiation risks for astronauts on multiple International Space Station missions. PLoS ONE.

[31] Cucinotta F.A., Alp M., Rowedder B., Kim M.-H.Y. (2015). Safe days in space with acceptable uncertainty from space radiation exposure. Life Sci. Space Res..

[32] Ozasa K., Grant E.J., Kodama K. (2018). Japanese Legacy Cohorts: The Life Span Study Atomic Bomb Survivor Cohort and Survivors’ Offspring. J. Epidemiol..

[33] Strigari L., Strolin S., Morganti A.G., Bartoloni A. (2021). Dose-Effects Models for Space Radiobiology: An Overview on Dose-Effect Relationships. Front. Public Health.

[34] Imaoka T. (2025). Trans-Scale Insights into Variability in Radiation Cancer Risk Across Tissues, Individuals, and Species. Biology.

[35] Hellweg C.E., Arena C., Baatout S., Baselet B., Beblo-Vranesevic K., Caplin N., Coos R., Da Pieve F., De Micco V., Foray N. (2023). Space Radiobiology. Radiobiology Textbook.

[36] Nguyen J., Moteabbed M., Paganetti H. (2015). Assessment of uncertainties in radiation-induced cancer risk predictions at clinically relevant doses. Med. Phys..

[37] Loffredo F., Vardaci E., Bianco D., Di Nitto A., Quarto M. (2023). Radioprotection for Astronauts’ Missions: Numerical Results on the Nomex Shielding Effectiveness. Life.

[38] Toto E., Lambertini L., Laurenzi S., Santonicola M.G. (2024). Recent Advances and Challenges in Polymer-Based Materials for Space Radiation Shielding. Polymers.

